# Survival benefits of antiretroviral therapy in Brazil: a model-based analysis

**DOI:** 10.7448/IAS.19.1.20623

**Published:** 2016-03-29

**Authors:** Paula M Luz, Michael P Girouard, Beatriz Grinsztejn, Kenneth A Freedberg, Valdilea G Veloso, Elena Losina, Claudio J Struchiner, Rachel L MacLean, Robert A Parker, A David Paltiel, Rochelle P Walensky

**Affiliations:** 1 The Instituto Nacional de Infectologia Evandro Chagas, Fundação Oswaldo Cruz, Rio de Janeiro, Brasil; 2 Medical Practice Evaluation Center, Massachusetts General Hospital Boston, MA, USA; 3 Division of General Internal Medicine, Massachusetts General Hospital Boston, MA, USA; 4 Division of Infectious Disease, Massachusetts General Hospital Boston, MA, USA; 5 Harvard University Center for AIDS Research, Harvard Medical School Boston, MA, USA; 6 Department of Epidemiology, Boston University School of Public Health Boston, MA, USA; 7 Department of Health Policy and Management, Harvard School of Public Health Boston, MA, USA; 8 Department of Orthopedic Surgery, Brigham and Women's Hospital Boston, MA, USA; 9 Department of Biostatistics, Boston University School of Public Health Boston, MA, USA; 10 Biostatistics Center, Massachusetts General Hospital Boston, MA, USA; 11 Department of Health Policy and Management, Yale School of Public Health, New Haven, CT, USA; 12 Division of Infectious Disease, Brigham and Women's Hospital Boston, MA, USA

**Keywords:** HIV, Brazil, highly active antiretroviral therapy, survival, modelling, Latin America

## Abstract

**Objective:**

In Brazil, universal provision of antiretroviral therapy (ART) has been guaranteed free of charge to eligible HIV-positive patients since December 1996. We sought to quantify the survival benefits of ART attributable to this programme.

**Methods:**

We used a previously published microsimulation model of HIV disease and treatment (CEPAC-International) and data from Brazil to estimate life expectancy increase for HIV-positive patients initiating ART in Brazil. We divided the period of 1997 to 2014 into six eras reflecting increased drug regimen efficacy, regimen availability and era-specific mean CD4 count at ART initiation. Patients were simulated first without ART and then with ART. The 2014-censored and lifetime survival benefits attributable to ART in each era were calculated as the product of the number of patients initiating ART in a given era and the increase in life expectancy attributable to ART in that era.

**Results:**

In total, we estimated that 598,741 individuals initiated ART. Projected life expectancy increased from 2.7, 3.3, 4.1, 4.9, 5.5 and 7.1 years without ART to 11.0, 17.5, 20.7, 23.0, 25.3, and 27.0 years with ART in Eras 1 through 6, respectively. Of the total projected lifetime survival benefit of 9.3 million life-years, 16% (or 1.5 million life-years) has been realized as of December 2014.

**Conclusions:**

Provision of ART through a national programme has led to dramatic survival benefits in Brazil, the majority of which are still to be realized. Improvements in initial and subsequent ART regimens and higher CD4 counts at ART initiation have contributed to these increasing benefits.

## Introduction

In 1996, the Brazilian government instituted the universal provision of combination antiretroviral therapy (ART), with virologic and immunologic monitoring, free of charge to eligible HIV-positive patients [1]. Along with these provisions, the Department of Sexually Transmitted Diseases, AIDS and Viral Hepatitis of the Brazilian Ministry of Health designated an expert panel to provide country-specific guidelines for the treatment of HIV-positive individuals. These guidelines have kept pace with widely accepted international standards of care for persons with HIV. In 1996, first-line ART consisted of zidovudine, lamivudine and indinavir, and individuals initiated ART upon presentation with an opportunistic infection (OI) or with CD4 < 200/µL. The most recent (2014) guidelines advocate treatment for all HIV-positive patients, irrespective of CD4 count, with a first-line regimen of tenofovir, lamivudine and efavirenz [2]; other therapies have been adopted for second-line and salvage regimens. Brazil's aggressive response to the HIV epidemic has been described as exemplary among low- and middle-income countries [3].

Even 20 years after its inception, the Brazilian universal ART treatment programme remains vulnerable. To secure continued public support for the programme, evidence of both its past benefit – and, more importantly, its likely lifesaving performance in the years ahead – is essential. Additionally, evidence of the success of the Brazilian ART experience could be influential in motivating expansion of ART programmes in other Latin American countries whose HIV epidemics closely resemble that in Brazil. Few epidemiological studies have quantified the impact of ART on population survival in Brazil over time. Early studies characterized pre-ART survival only [4,5]. Studies from the late 1990s and early 2000s characterized survival for subsets of patients [6–12] or with alternative AIDS definitions [13]. Although no analysis could portray the past counterfactual in all its detail nor portray the future course of the epidemic with precision, our objective was to offer a credible window on both. We used a model-based approach to quantify the 2014-censored and projected lifetime survival benefits achieved with the incremental improvements of the Brazilian HIV treatment programme.

## Methods

### Analytic overview

We used a previously published microsimulation model of HIV disease and treatment [14–18], the Cost-Effectiveness of Preventing AIDS Complications-International (CEPAC-I) model, using data from Brazil [18] to project the survival benefits attributable to provision of ART by the Brazilian national HIV treatment programme for patients initiating ART from 1997 to 2014. We divided this time frame into six eras, reflecting improvements in drug regimen efficacy and availability over time, as reported in the Brazilian HIV Treatment Guidelines [2]: Era 1 (1997 to 1999), Era 2 (2000 to 2003), Era 3 (2004 to 2007), Era 4 (2008 to 2012), Era 5 (2013) and Era 6 (2014). ART eligibility criteria also changed over time, which we modelled by varying cohort characteristics, including CD4 count at ART initiation. Annual cohorts of patients with characteristics reflective of their respective eras were simulated from treatment initiation in that year under two scenarios: without ART and with ART. To limit the analysis to the benefit of ART, both the treatment and no-treatment scenarios included the use of guideline-concordant OI prophylaxis. Life expectancy censored at 2014 for each cohort was recorded. Increase in life expectancy was calculated for each era and then multiplied by the projected number of patients initiating ART in that era to obtain survival benefits. Results were extended to uncensored life expectancy to determine the projected lifetime survival benefits of ART and to estimate the proportion of benefits that has already been realized. Median and 20-year survival were estimated for each era for the two scenarios, without ART and with ART.

### The CEPAC-I model

CEPAC-I is an individual patient-level microsimulation model of HIV natural history, disease and treatment. Simulated patients are randomly generated from user-specifiable cohort characteristic distributions and are subject to treatment regimens and HIV monitoring policies defined by the model user. HIV disease progression, defined as immune system deterioration, onset and relapse of OIs, and mortality, is influenced by CD4 count, HIV viral load and OI history. ART use decreases morbidity and mortality through viral suppression and CD4 count increase. OI incidence, OI mortality and AIDS-related mortality are stratified by a patient's CD4 count.

Patients eligible for ART are assigned a level of treatment adherence drawn from a distribution of pharmacy withdrawal records at an HIV/AIDS reference centre in São Paulo, Brazil [19]. Initial suppression on ART and subsequent virologic failure rates are dependent upon a patient's level of adherence [20], with higher adherence corresponding to higher likelihood of virologic suppression. Following the Brazilian standard of care, patients on ART receive CD4 and HIV RNA monitoring every six months [2]. After regimen failure – defined as a WHO Stage 3 or 4 OI or a confirmed detectable viral load – has been detected, patients switch to a subsequent line of ART, which is defined by regimen availability in Brazil at the time of failure for any given era. After failing the last available line, patients remain on their final ART regimen until death. Patients are also subject to a monthly, adherence-dependent probability of becoming lost to follow-up. Those lost are subject to virologic rebound and HIV disease natural history; they also have a monthly probability of returning to care. Cohorts of one million patients are simulated to achieve stable per-person estimates of survival and clinical events. A more complete description of the CEPAC-I model can be found in the Supplementary file and in previous publications [18,20,21] or in the *CEPAC Model User's Guide* [22].

### Input parameters

Model parameters for cohort characteristics and natural history were derived from the HIV Clinical Cohort at the Instituto Nacional de Infectologia Evandro Chagas (INI), Fundação Oswaldo Cruz, and from published Brazilian governmental data. INI is a public research and healthcare institution situated in Rio de Janeiro, Brazil, and is one of Brazil's largest reference centres for HIV research and treatment. It has provided care to over 5500 HIV-positive patients in the Rio de Janeiro metropolitan area since 1986; we had access to client-level data for model parameterization [23–25].

#### Cohort characteristics

Characteristics of the simulated annual cohorts were derived from the 2290 treatment-naïve HIV-positive adults initiating ART at INI between 2000 and 2014. Mean (SD) age was 37 (10) years, and 70% of patients were male (Table 1) with negligible variation by era. In contrast, other parameters varied by era, including CD4 count at ART initiation, which ranged from 185/µL (Era 1) to 518/µL (Era 6), and the fraction of patients with an OI history (i.e. a diagnosis of AIDS) at ART initiation (see Supplementary Figures 1 and 2 for additional details).

**Table 1:** Select model parameter inputs

Model parameter	Base case value	Ranges examined	Reference
**Cohort characteristics**			INI
Age (SD), (years)	37 (10)	27–47	
Male sex, (%)	70	60–80	
Mean (SD) CD4 at ART initiation, (/µL)			
Era 1	185 (123)	92–277	
Era 2	231 (154)	116–347	
Era 3	284 (189)	142–426	
Era 4	343 (229)	172–515	
Era 5	383 (255)	192–575	
Era 6	518 (273)	259–777	
Proportion of patients with OI history at ART initiation, (%)			
Era 1	63.7	31.9–95.6	
Era 2	54.0	27.0–81.0	
Era 3	42.5	21.3–63.8	
Era 4	30.7	15.3–46.0	
Era 5	23.7	11.9–35.6	
Era 6	13.0	6.5–19.5	
HIV RNA level at ART initiation, all Eras			
> 100,000	45.1	–	
30,001–100,000	25.7	–	
10,001–30,000	18.3	–	
3001–10,000	10.9	–	
≤ 3000	0.0	–	
**Disease natural history**			INI
Monthly risk of chronic AIDS death*, (%)			
Without history of OI	0.82–0.02	–	
With history of OI	5.67–0.02	–	
**Antiretroviral therapy**			
Adherence distribution			[19]
> 95%	38.9	19.5–58.4	
80–95%	30.2	–	
< 80%	30.9	–	
First-line virologic suppression at six months, (%)			
Era 1	60	40–80	[30]
Eras 2–4	80	70–90	[38]
Eras 5–6	90	85–95	[38]
Virologic failure rate, (/100PM)			
Currently prescribed regimens	0.16	0.08–0.24	[40,41]
Formerly prescribed regimens	3.96	1.98–5.94	[42]
Rate of loss to follow-up, (/1000PY)	10.1	5.1–15.2	[43]
Rate of return to care, (/1000PY)	818	409–1227	INI

SD: standard deviation; INI: Instituto Nacional de Infectologia Evandro Chagas; OI: opportunistic infection; PM: person-months; PY: person-years.

* Risks are stratified by CD4 count, with higher risk associated with lower CD4 count.

#### Natural history

Natural history was derived from a study sample that included adult patients (≥18 years) who enrolled in the INI cohort from 1986 through 2010. These patients were followed for a minimum of 60 days post-enrolment. Cohort procedures include several means of checking patient's vital status to minimize the risk of bias when reporting on mortality (see Supplementary file for details). Natural history parameters included incidence and mortality rates of OIs, both stratified by CD4 count (but not by age and gender); these parameters were assumed to remain constant for the time period evaluated and were converted into monthly probabilities for model input [18]. Brazil-specific non-AIDS mortality probabilities were age- and gender-stratified from the life tables of the United Nations Department of Economic and Social Affairs [26]. To account for lower life expectancy for HIV-positive individuals attributable to risk factors associated with HIV infection (e.g. injection drug use), non-AIDS mortality rates were adjusted by standardized mortality ratios corresponding to various HIV risk groups [27]. Rates of off-ART CD4 decline were stratified by CD4 count and HIV RNA and obtained from an analysis of two multicentre cohorts of untreated HIV-positive patients [28,29]. We used these natural history parameters to simulate survival for patients without ART.

#### ART efficacy and availability

Per universal access in Brazil [1], the model assumes universal and era-dependent access to every available ART regimen, including subsequent lines of therapy. The cohort adherence distribution was from a study of ART pharmacy withdrawals in São Paulo, Brazil, and was assumed to be constant across all eras; mean adherence was 83.5%, meaning that the average patient was in possession of 83.5% of his or her prescribed medication in the previous 12 months [19]. ART regimen sequencing and availability varied by era; patients in earlier eras who survived to subsequent eras skipped obsolete regimens (Table 2) and switched to newer, more effective regimens upon virologic failure. Rates of virologic suppression and CD4 count increase for suppressed patients were derived from clinical trials [30–39]. Virologic suppression for first-line ART at 24 weeks, which was dependent on adherence, ranged from an average of 60% (Era 1) to 90% (Eras 5 to 6). The rate of later virologic failure for those who achieved suppression, also dependent on adherence, averaged 0.16/100PM for regimens currently prescribed in Brazil [40,41] and 3.96/100PM for older regimens no longer in use [42].

**Table 2:** ART regimen sequencing and efficacy by era

Era		Regimen	Year regimen available	Proportion of patients achieving virologic suppression at 24 weeks^a^	Estimated CD4 increase at 12 months for suppressed patients (cells/µL)	Reference
**ERA 1 (1997–1999)**	**1)**	**AZT + 3TC + IDV**	1997	60	140	[30]
	**2)**	Alternative regimens after PI failure, w/o genotype*	1997	22^b^	100	[31]
	3)	EFV + 2NRTIs (3TC/d4T or 3TC/ddI or d4T/ddI)	2000	62	140	[32]
	4)	LPV/r + 2NRTIs*	2000	62	100	[33]
	5)	ATV/r + TDF + 1NRTI	2004	65	110	[33]
	6)	ENF + optimized background*	2006	30	140	[34]
	7)	RAL + DRV/r + 2NRTIs	2008	75	150	[35]
	8)	ETV + PI/r + 2NRTIs	2013	70	150	[36]
	9)	CCR5 + PI/r + 2NRTIs	2013	60	140	[37]
**ERA 2 (2000–2003)**	**1)**	**EFV + AZT + 3TC**	2000	80	180	[38]
	**2)**	LPV/R + 2NRTIs*	2000	62	100	[33]
	**3)**	Alternative regimens after PI failure, with genotype*	2000	34^b^	90	[31]
	4)	ATV/r + TDF + 1NRTI	2004	65	110	[33]
	5)	ENF + optimized background*	2006	30	140	[34]
	6)	RAL + DRV/r + 2NRTIs	2008	75	150	[35]
	7)	ETV + PI/r + 2NRTIs	2013	70	150	[36]
	8)	CCR5 + PI/r + 2NRTIs	2013	60	140	[37]
**ERA 3 (2004–2007)**	**1)**	**EFV + AZT + 3TC**	2004	80	180	[38]
	**2)**	**LPV/r + 2NRTIs**	2004	62	100	[33]
	**3)**	**ATV/r + TDF + 1NRTI**	2004	65	110	[33]
	4)	ENF + optimized background*	2006	30	140	[34]
	5)	RAL + DRV/r + 2NRTIs	2008	75	150	[35]
	6)	ETV + PI/r + 2NRTIs	2013	70	150	[36]
	7)	CCR5 + PI/r + 2NRTIs	2013	60	140	[37]
**ERA 4 (2008–2012)**	**1)**	**EFV + AZT + 3TC**	2008	80	180	[38]
	**2)**	LPV/r + 2NRTIs*	2008	62	100	[33]
	**3)**	**ATV/r + TDF + 1NRTI**	2008	80	120	[39]
	**4)**	**RAL + DRV/r + 2NRTIs**	2008	75	150	[35]
	**5)**	ENF + optimized background*	2008	30	140	[34]
	6)	ETV + PI/r + 2NRTIs	2013	70	150	[36]
	7)	CCR5 + PI/r + 2NRTIs	2013	60	140	[37]
**ERA 5 (2013)**	**1)**	**TDF + FTC + EFV**	2013	90	200	[38]
	**2)**	**ATV/r + 2NRTIs**	2013	80	130	[39]
	**3)**	**RAL + DRV/r + 2NRTIs**	2013	75	150	[35]
	**4)**	**ETV + PI/r + 2NRTIs**	2013	70	150	[36]
	**5)**	**CCR5 + PI/r + 2NRTIs**	2013	60	140	[37]
**ERA 6 (2014)**	**1)**	**TDF + FTC + EFV**	2014	90	200	[38]
	**2)**	**ATV/r + 2NRTIs**	2014	80	130	[39]
	**3)**	**RAL + DRV/r + 2NRTIs**	2014	75	150	[35]
	**4)**	**ETV + PI/r + 2NRTIs**	2014	70	150	[36]
	**5)**	**CCR5 + PI/r + 2NRTIs**	2014	60	140	[37]

HVL: HIV viral load; AZT: zidovudine; 3TC: lamivudine; IDV: indinavir; PI: protease inhibitor; EFV: efavirenz; NRTI: nucleoside reverse transcriptase inhibitor, d4T: stavudine; ddI: didanosine; LPV: lopinavir; r: boosted ritonavir; ATV: atazanavir; TDF: tenofovir; FTC: emtricitabine; ENF: enfurvitide; RAL: raltegravir; DRV: darunavir; ETV: etravirine; CCR5: C-C chemokine receptor type 5.

a Virologic suppression defined as HVL < 400 or <500 copies/mL, depending on the source

b suppression was assessed at 12 weeks because of reporting in the source.

* Indicates a regimen that will be skipped if a subsequent, more effective regimen is available.

Bolded regimens are those available at the beginning of each era.

#### Loss to follow-up and return to care

The loss to follow-up rate of 10.1/1000 person-years (PY) was derived from the HIV-Brazil Cohort Study [43], a nationwide, multicentre study of over 5000 patients receiving ART from 2003 to 2010. The rate of return to care (818/1000PY) was from the INI clinical cohort.

### Estimates of number initiating ART

The Brazilian Ministry of Health reports the annual number of patients receiving ART through the national ART programme from 1999 to 2012 [27]. We created two linear models by extrapolating backward two years (1997 and 1998) and forward two years (2013 and 2014) to obtain estimates for the entire time frame of interest, 1997 to 2014 (see Supplementary Figure 3). We assumed that all patients on ART in 1997 (60,316) initiated ART that year. Since the number of patients initiating ART in each year is not available, we calculated this as the reported number of patients on ART in that year minus the number of patients on ART from the previous year who survived and were not lost to follow-up according to CEPAC-I model survival output, a methodology used in a previous survival benefits analysis [44].

### 2014-censored and lifetime survival benefits

Per-person survival benefits for each era were calculated as the difference in 2014-censored life expectancy for patients on ART in a given era and that of patients without ART in that era. This means that patients in the 1997 cohort accrued a maximum of 18 years of survival through 2014, whereas those in the 2014 cohort accrued a maximum of only one year. To obtain total cohort benefits for a given era, we multiplied the per-person benefits by the number of patients initiating ART in that era. Survival benefits were summed across all eras to obtain total population estimates of survival benefits attributable to the Brazilian national ART programme. In addition to 2014-censored results, uncensored survival benefits results were calculated in the same way to estimate projected lifetime benefits of the Brazilian national ART programme for these patients and to estimate the proportion of survival benefits that has not yet been realized.

### Sensitivity analyses

We varied uncertain model parameters across a plausible range (Table 1) using the number of patients initiating ART in each era to evaluate the robustness of the survival benefit estimates. These parameters, varied in all eras, included the following: mean CD4 count at ART initiation, proportion of patients with greater than 95% adherence, ART failure at six months for all ART lines, proportion of patients initiating ART with a history of OI, virologic failure rate for suppressed patients, loss to follow-up rate, return to care rate for patients lost to follow-up. All parameters were varied from 50 to 150% of base case values.

In multivariate sensitivity analysis, we created best-case and worst-case scenarios, using the most and least optimistic parameter assumptions (50 or 150% of the base case values). In the worst-case scenario, we assumed decreased mean CD4 count at ART initiation, increased proportion of patients initiating ART with a history of OI, increased ART failure at six months, increased virologic failure rate for suppressed patients, increased loss to follow-up rate and decreased return to care rate for patients lost to follow-up. The opposite was assumed for the best-case scenario. Adherence, since it affects ART failure at six months, virologic failure for suppressed patients and loss to follow-up, was not modified in the best-case and worst-case scenarios to avoid double counting effects.

## Results

### Base case

The number of patients initiating ART in each of the six eras was 114,062, 115,363, 92,895, 189,741, 43,755, and 42,925, respectively, for a total of 598,741 patients initiating ART between 1997 and 2014 (Table 3). Results censored in December 2014 demonstrate that Era 1 yielded a life expectancy of 2.7 years without ART compared to 6.5 years with ART, a per-person increase of 3.8 years (Table 3, columns B–D). Survival increased in those without ART owing to higher CD4 counts at presentation as the eras progressed. To understand this, note that each era is characterized not only by improved treatment regimens but also by earlier presentation to care and treatment initiation. Consequently, patients have higher mean CD4 counts at simulation entry – even those who do not receive ART. Survival benefits censored at 2014 for subsequent eras [2–6] decreased as the eras progressed due to the shorter time horizon for survival accrual relative to the earlier eras and due to longer survival without ART. The sum of products of the number of patients initiating ART and the 2014-censored per-person survival benefits yielded an estimated 1.5 million life-years saved as of December 2014; approximately 87% of this benefit was accrued by patients initiating ART in the first three eras.

**Table 3:** 2014-censored and lifetime survival benefits of the Brazilian national ART program for patients starting ART between 1997 and 2014

	2014 censored results (LY)					Lifetime results (LY)			
		
Era	A: Persons initiating ART^a^	B: Per capita life expectancy, without ART	C: Per capita life expectancy, with ART^b^	D: Per capita survival benefit^c^ [C–B]	E: Survival benefit [A×D]	B’: Per capita life expectancy, without ART^d^	C’: Per capita life expectancy, with ART	D’: Per capita survival benefit [C’-B’]	E’: Survival benefit [A×D’]
Era 1 (1997–1999)	114,062	2.7	6.5	3.8	433,436	2.7	11.0	8.3	946,715
Era 2 (2000–2003)	115,363	3.2	8.1	4.9	565,279	3.3	17.5	14.2	1,638,155
Era 3 (2004–2007)	92,895	3.7	7.0	3.3	306,554	4.1	20.7	16.6	1,542,057
Era 4 (2008–2012)	189,741	3.3	4.3	1.0	189,741	4.9	23.0	18.1	3,434,312
Era 5 (2013)	43,755	1.8	1.9	0.1	4375	5.5	25.3	19.8	866,349
Era 6 (2014)	42,925	1.0	1.0	0.0*	385	7.1	27.0	19.9	854,208
Total	598,741				1,499,770				9,281,796

a Calculated as the number of patients on ART in the given era minus the patients still alive from previous eras

b censored life expectancy for 1997 cohort is out of a possible 18 years whereas that for 2014 is out of a possible one year

c calculated by subtracting life expectancies during 1997–2014 for the Without ART simulations (column B) from those for the ART simulations (column C). Survival gains in 1997 are thus measured over 18 years, whereas those in 2014 are measured over one year

d increase in life expectancy without ART is due to an increase in CD4 count at presentation by era.

* Value is non-zero but is reported as zero due to rounding.

**Figure 1:**
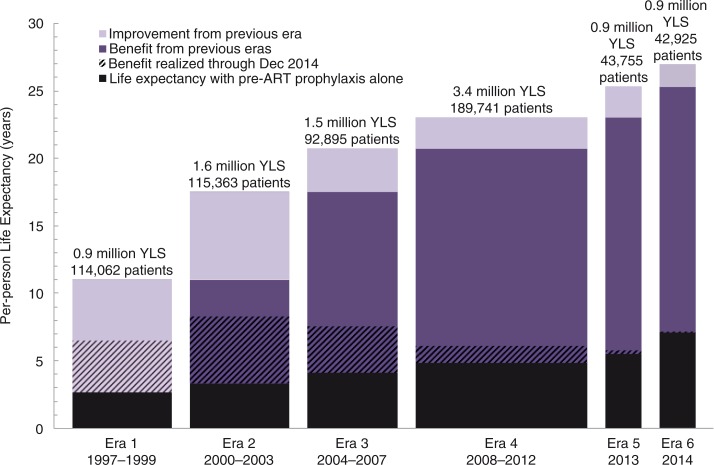
Years of life saved per-person in each era produced by model simulations. Bar width corresponds to the number of patients in each era and total coloured area corresponds to lifetime survival benefits. Survival benefits realized as of December 2014 are shaded with diagonal lines. YLS: years of life saved; ART: antiretroviral therapy.

For lifetime outcomes, Era 1 had a life expectancy of 2.7 years without ART compared to 11.0 years with ART, for a per-person increase of 8.3 years (Table 3, columns B′–D′). Per-person survival benefits for subsequent eras were even greater, ranging from 14.2 years for Era 2 to 19.9 years for Era 6. Per-person life expectancy in each era is illustrated in Figure 1, as is the survival benefit attributable to ART availability in each era compared to no ART and compared to the era immediately before. Increased per-person survival yielded a total population anticipated lifetime survival benefit of 9.3 million life-years, 7.8 million (84%) of which have yet to be realized (Figure 1).

**Figure 2:**
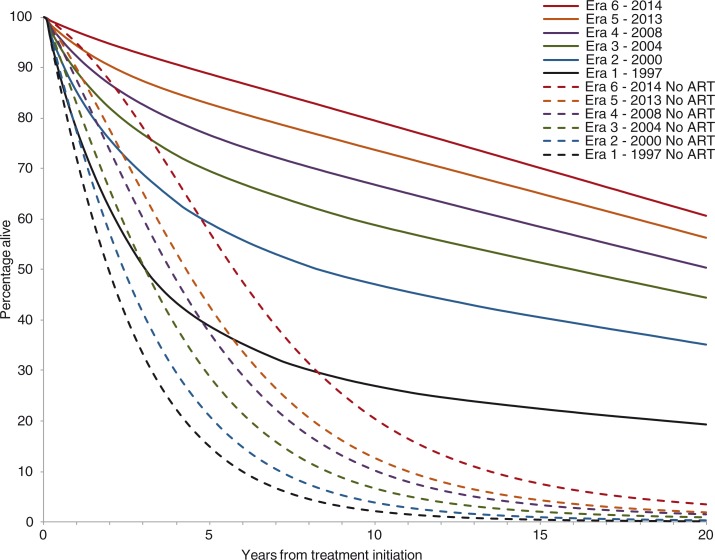
Survival curve over 20 years from treatment initiation for each era of ART in Brazil. Survival with ART is displayed in solid lines, and survival without ART is displayed in dotted lines. ART: antiretroviral therapy.

Twenty-year survival, both without and with ART, increased in each subsequent era (Figure 2, Table 4). In the without-ART analyses (dashed), this resulted from increased CD4 counts at ART initiation and the decreased proportion of patients presenting with OIs. Twenty-year survival for patients in Era 1 increased from 0.2% without ART to 20.8% with ART; median survival for these patients increased from 2.0 years without ART to 3.3 years with ART (see Figure 2 and Table 4). In Era 6, 20-year survival increased from 3.4% without ART to 60.6% with ART; median survival for these patients increased from 5.7 years without ART to 25.7 years with ART.

**Table 4:** Predicted median survival and 20-year survival for all six eras, without and with ART

Era	Median survival, without ART (years)	Median survival, with ART (years)	Predicted proportion alive at 20 years, without ART (%)	Predicted proportion alive at 20 years, with ART (%)
Era 1 (1997–1999)	2.0	3.3	0.2	20.8
Era 2 (2000–2003)	2.4	9.5	0.4	36.9
Era 3 (2004–2007)	3.0	16.4	0.8	45.0
Era 4 (2008–2012)	3.8	20.4	1.4	50.6
Era 5 (2013)	4.3	23.7	1.9	56.4
Era 6 (2014)	5.7	25.7	3.4	60.6

### Sensitivity analyses

**Figure 3:**
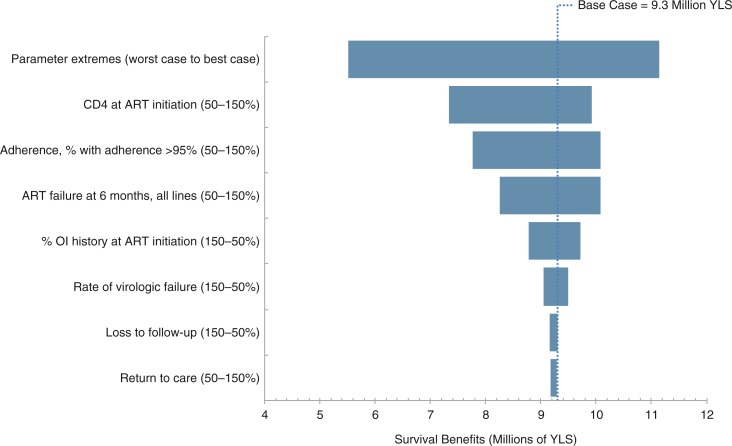
Tornado diagram of one-way sensitivity analyses of lifetime survival benefits of ART in Brazil. Each horizontal bar represents the range of survival benefits produced by varying a given model parameter across the ranges in parentheses. The vertical line represents the base case.

Projected lifetime survival benefits were generally stable across the ranges of parameters examined (Figure 3). The most influential parameter was mean CD4 count at ART initiation for all eras; decreasing or increasing this by a relative 50% gave lifetime survival benefit estimates of 7.3 and 9.9 million life-years. Other influential parameters included the proportion of patients with >95% adherence (7.8 to 10.1 million life-years), ART failure at six months for all regimens (8.3 to 10.1 million life-years) and the percentage of patients starting ART with a history of OIs (8.8 to 9.7 million life-years). Rate of loss to follow-up and return to care did not substantially influence lifetime survival benefits. In the worst-case scenario, lifetime survival benefits were estimated at 5.5 million life-years. The best-case scenario yielded projected lifetime survival benefits of 11.1 million life-years.

## Discussion

Based on reporting by the Brazilian Ministry of Health [27], we estimated that almost 600,000 individuals have received ART, free of charge, in Brazil between 1997 and 2014. Our results predict that the lifetime survival benefits achievable by these individuals will reach 9.3 million life-years. Of this total, we found that 16% of the benefits have been realized as of December 2014 (1.5 million life-years), whereas 7.8 million life-years have yet to be realized. Our results also predict that lifetime per-person survival benefits surpass a decade of life for Era 2 (spanning 2000 to 2004) and that these individual benefits for Era 3 and beyond are even greater, >16 additional years of life. These results, which corroborate the literature on the survival benefits of ART in Brazil [6–11,13], provide a comprehensive view of the health benefits accrued through the Brazilian HIV treatment programme.

We have structured this analysis to portray the regimens used in Brazil and incorporated country-specific values for the prevalence of AIDS-related infections, late presentation to care and suboptimal adherence, all of which conservatively reduce survival benefits. Additionally, to restrict the calculation of survival benefits to only those conferred by ART, we incorporated other important facets of the Brazilian response to the HIV epidemic, including prophylaxis and comprehensive OI care, when modelling the eras without ART. To the extent that the simulations without and with ART were similar in all aspects except for ART (i.e. treatment adherence, clinical care, among other parameters), we can infer that the benefits reported here derive solely from the use of ART. Moreover, per-person life expectancy increases in subsequent eras can be attributed to improved ART regimens as drugs with increased efficacy were incorporated.

Currently, the HIV epidemic of other Latin American countries resembles that of Brazil. Argentina, Chile, Mexico, Peru and Venezuela have similarly concentrated epidemics, with an HIV prevalence in the general population below 1%. In these countries, the proportion of eligible HIV-positive individuals on ART is similar to that reported in Brazil (estimated from 60 to 86%, with a region-wide estimate of 75%; see Supplementary Table 2). This suggests that survival benefits for the recent eras reported in this paper might be generalizable to other countries in the region with similar ART guidelines. Nevertheless, care is needed when extrapolating this achievement, given that Brazil's HIV treatment programme dates to 1996, whereas most other Latin American countries, such as Mexico, Peru and Chile, initiated national-level treatment programmes in 2001, 2004 and 2005, respectively [45–47]. Further, specific enhancements such as treatment monitoring (incorporated in Brazil in conjunction with HIV treatment), genotype resistance testing after failure (started in Brazil in early 2000) and availability of third-line and salvage regimens should be acknowledged, as these are often not uniformly available and can influence the applicability of our results to other settings.

This analysis has several limitations. A single source was used for parameters such as mean CD4 count at ART initiation, proportion of patients initiating ART with a history of OI, medication adherence, loss to follow up rate and return-to-care rate for patients previously lost. We acknowledge that input parameters from a single site may not always be representative of the entire country and have used sensitivity analyses to demonstrate where variation would have a major impact on results. For example, we used pharmacy withdrawal information from a study in São Paulo as a proxy for medication adherence country-wide. Because adherence in the São Paulo study may not reflect the country as a whole – and because pharmacy withdrawals may not correlate perfectly with actual adherence –we employed sensitivity analysis to consider the impact of varying this parameter on outcomes; when adherence was 50 and 150% of the base case, survival benefits changed to 7.8 and 10.1 million life-years, respectively. Next, although we did not adjust the model for risk group, age or sex, we did compare similar cohort compositions both with and without ART to achieve survival benefit totals; as such, this limitation should not greatly influence the present analysis. Further, our analysis did not incorporate regional and other demographic variation in mortality and other key model parameters that might more accurately reflect Brazil's heterogeneity; thus, our analysis cannot speak to variation in survival on or off ART that may result from underlying life expectancy differences between regions. Additionally, though we did not explicitly model changes in Brazil's ART initiation criteria, we modelled the evolution of the Brazilian HIV Treatment Guidelines as closely as possible with respect to drugs and regimens and conservatively assumed no improvements in treatment or care in our projected lifetime results. Thus, our results are likely consistent with ART initiation criteria since, in general, patients initiated ART later than recommended in Brazil in all eras [48]. Importantly, our results exclude the benefits attributable to the prevention of mother-to-child transmission, the treatment of children and adolescents and the reduction of horizontal HIV transmission due to suppressed viral load. Our results also exclude the economic returns of ART treatment [49]. Furthermore, we did not explicitly model expansion of HIV prevention, testing and treatment infrastructure. Finally, the current version of the CEPAC-I model does not include long-term co-morbidities related to ART exposure. Including these conditions may reduce the projected survival benefits of ART.

## Conclusions

Over the past 20 years, Brazil's national programme of free ART access to patients has led to dramatic survival benefits, the vast majority of which are still to be realized. Higher CD4 counts at treatment initiation and improvements in initial and subsequent ART regimens have all contributed substantially to these benefits. Increased HIV testing, with even earlier ART initiation and improved rates of linkage to care, as well as interventions to improve ART adherence, could lead to additional survival benefits in the future.

## Supplementary Material

Survival benefits of antiretroviral therapy in Brazil: a model-based analysis
