# Supplementary material for: Survival benefits of antiretroviral therapy in Brazil: a model-based analysis
Source: J Int AIDS Soc. 2016 Mar 29;19(1):20623. doi: 10.7448/IAS.19.1.20623 (PMC4814587; doi:10.7448/IAS.19.1.20623)
Supplement: Survival benefits of antiretroviral therapy in Brazil: a model-based analysis [file JIAS-19-20623-s001.pdf]

Supplemental Digital Content 1

Survival Benefits of Antiretroviral Therapy in Brazil

Luz et al.

In areas where computer programming and methods are identical, the text from this appendix is similar to the online technical appendix available from:

Luz PM, Morris BL, Grinsztejn B, Freedberg KA, Veloso VG, Walensky RP, *et al*. Cost-effectiveness of genotype testing for primary resistance in Brazil. *J Acquir Immune Defic Syndr* 2015, **68**: 152-61.

CEPAC International Model

Overview

The CEPAC International Model is a computer-based, state-transition, Monte Carlo simulation model of the progression and outcomes of HIV disease in a hypothetical cohort of patients. "State-transition" means that the model characterizes the natural history of illness in an individual patient as a sequence of monthly transitions from one "health state" to another. "Monte Carlo" refers to a random number generator and set of estimated probabilities that are used to determine the sequence of movements between health states for a particular patient. Each individual patient's clinical course is followed from the time of entry into the model until death. A running tally is maintained of all clinical events and the length of time spent in each health state. Upon the patient's death, summary statistics are recorded and a new patient enters the model. This process is then repeated for a large number of simulated patients (statistical convergence can typically be achieved with cohort sizes of one million), at which point overall performance measures, such as average life expectancy, are computed.

Health States

In the Disease Model, health states are chosen to be descriptive of the patient's current health, relevant history, and resource utilization patterns. They are designed to be predictive of clinical prognosis, including disease progression, immune system deterioration, development and relapse of different opportunistic infections (OIs), toxic reactions to medications, resistance to therapy, and mortality. The model defines general categories of health states: chronic infection, acute complication, and death. Most of the time, patients are in one of the chronic states, where progression of disease and immune system deterioration (CD4 decline) take place. Patients who develop an acute complication (e.g., an OI or drug-related toxicity) temporarily move to an acute health state, where both resource consumption levels and mortality rates are higher. Deaths can occur from either a chronic or an acute state and can be attributed to a particular OI, chronic AIDS (e.g., wasting), or non-AIDS-related causes.

The chronic and acute health states are stratified by: actual current and nadir CD4 count (>500/µl; 301–500/µl; 201–300/µl; 101–200/µl; 51–100/µl; and 0-50/µl) and current and set-point HIV RNA level (>100,000 copies/mL, 30,001-100,000 copies/mL; 10,001–30,000 copies/ mL; 3,001–10,000 copies/ mL; 501–3,000 copies/ mL; 51-500 copies/ mL; 0-50 copies/mL). Drawing from distributions of patient characteristics (age, sex, CD4 count and HIV RNA level) derived from the INI Cohort (see below), a patient is randomly assigned to a health state upon model entry. By permitting the user to define initial population distributions for patient age, sex, CD4 count, HIV RNA, and other demographic and clinical attributes, the model has the flexibility to explore a broad range of different patient cohorts, which we have used to represent the characteristics of patients initiating ART in each of the six eras.

At the start of each one-month cycle, the model records the patient's CD4 count, HIV RNA level, history of acute illness, and current therapies and uses these characteristics to determine the probabilities that indicate movement to a new state in the subsequent month. Monthly probabilities of events are estimated directly from the INI cohort and published data. The model treats HIV RNA as the primary driver of immune system deterioration, and thus the assigned viral load level determines the rate at which the patient's CD4 count will decline in the absence of ART (1).

We are careful to distinguish in the model "actual" CD4 count and HIV RNA – i.e. the underlying immunologic and virologic state, regardless of whether it is measured by a laboratory test– from "observed" CD4 count and HIV RNA – that which is measured by a test and upon which clinical decisions can be made. Clinical events within the model are predicated on patient's "actual" CD4 count and viral load status, while treatment decisions remain based on "observed" CD4 count and viral load status.

Clinical Visits and Laboratory Monitoring

Upon entry to the model, all patients undergo a clinic visit to observe their initial OI histories. CD4/HIV RNA monitoring also occur at model entry. At this initial visit, if

specific criteria are met, patients will initiate prophylaxis and antiretroviral therapies. Subsequent clinic visits will then be scheduled at regularly specified intervals to determine ART eligibility criteria and to monitor treatment success or failure. In addition to the regularly scheduled clinic visits, certain events (OIs) may trigger an emergency clinic visit to occur in that month. An emergency clinic visit is associated with the same clinical decision-making opportunities as a routine visit, and at most one clinic visit can occur in a given month.

Because CD4 and/or HIV RNA monitoring is available, these tests are generally administered at the time of a clinic visit. Standard testing frequency may be user-defined (in Brazil, every 6 months); other conditions may also trigger additional tests, including observed ART failures that require confirmation by CD4 or viral load, depending on the confirmation method specified by the user.

ART and ART Efficacy

The model has capacity to simulate up to ten lines of ART per cohort simulated, administered sequentially. We limit this analysis to patients initiating ART in Brazil. All patients begin ART upon entering the model. ART regimens are available to patients experiencing virologic failure in a way that mirrors the historical chronology of ART availability in Brazil. For example, patients initiating ART in 1997 (Era 1) initially have two lines of ART available, an initial zidovudine/indinavir/lamivudine regimen and one salvage regimen (Table 2, main text). Patients from this era who survive to the year 2000 (Era 2) can switch to an efavirenz-based regimen upon virologic failure, as efavirenz became available in Brazil in that year. These patients who survive to the year 2000 can also benefit from a PI-based second-line regimen upon failing an efavirenz-based regimen before moving on to a subsequent salvage regimen.

The model's handling of efficacy and durability of antiretroviral therapy depends on a patient's "adherence" level (0-100%), drawn at ART initiation from a logit distribution based on pharmacy refill data. We estimate ART efficacy from data on viral suppression

and CD4 count change over time, as reported in clinical trials. From these data, we derive a probability of “early” (within 6 months) and “late” (beyond 6 months) failure for each antiretroviral regimen to be considered; patients with higher adherence values experience lower probability of both early and late failure. This early/late failure structure allows patients to transition from virologic suppression to “failure” with the appropriate change in HIV RNA and CD4 count. Virologic suppression results in CD4 increases, also in these two time phases, that occur in concordance with data reported in clinical trials. An initial large CD4 benefit occurs in the “early” period, followed by a modest benefit that occurs over a longer time horizon, as long as the patient remains virologically suppressed.

Patients may be lost to follow-up while on ART; those that are lost to follow-up may return to care and resume ART. A patient is evaluated for stopping or switching the ART regimen at every clinic visit. The criteria for regimen change can be specified differently for each individual regimen. Upon meeting criteria for ART failure and switching, the patient is started on the next available regimen as specified by the user. If a more effective subsequent regimen is available at the time of virologic failure, a patient can skip a less effective regimen and move directly to the more effective regimen.

The model makes a distinction between patients actually failing an ART regimen and those who are observed to fail a regimen. The former can be regarded as patients in whom therapy stops providing any substantive biological benefit to the patient. The latter simulates the clinical observation of a new OI, or laboratory detection of CD4 decline or viral load increase, indicating a regimen’s lack of continued benefit, at which point the patients may be taken off that regimen. Upon laboratory-observed and confirmed failure – defined by HIV RNA>500 copies/ml or CD4 count decrease >25% – patients are initiated on a subsequent-line regimen, until no others are available. At that point, patients remain on their final regimen until death.

Monthly Cycle of the Model

Because all events in the program occur discretely, it is important to keep in mind the order of evaluation in each month of a simulated patient. Taking all the mechanisms described above together for HIV-infected patients, each regular monthly cycle in the program involve the following steps in order:

1. increase the patient's age, in months
2. update the patient's true CD4 and HIV RNA for the month
3. for all prophylaxes the patient is currently on, see if an associated toxic event occurs
4. if the patient is on an ART regimen, see if an associated toxic event occurs
    a. if an ART major toxicity event occurred, see if it also caused death – if death did occur, stop the simulation of the patient
5. determine if the patient dies from chronic AIDS or non-AIDS causes
    a. if death occurs, stop the simulation of the patient
6. determine if an acute OI event will occur this month
    a. if an OI event occurs, see if it also causes death from the OI and thus stop the simulation of the patient
    b. if the patient is not detected as HIV+, make the patient detected
7. compute whether a CD4 and/or HVL test(s) should be performed this month, based on last tests
8. if a CD4 test is to be performed, do so
    a. start and stop OI prophylaxes as necessary based on the patient's new observed CD4
9. if a viral load test is to be performed, do so
    a. if the patient is on an ART, see if the new observed viral load (and CD4) results in a failed ART diagnosis
        i. if the number of repeat failure diagnoses is enough for a confirmed failure, take the patient off the ART regimen
        ii. if the patient has not actually failed the ART regimen (i.e. the failure diagnoses are incorrect), treat the patient's CD4 and HIV RNA

levels as in ART failure to reflect the discontinuation of the effective therapy

10. if the patient is not on any ART regimen and qualifies for a new line of ART (per observed CD4 and HIV RNA, number of lag months between ARTs, etc.), start the patient on the next regimen
11. for each prophylaxis the patient is on, determine if resistance is to start in the current month
12. update the patient's accumulated life months and quality adjusted life months

Description of the INI HIV Clinical Cohort

The Laboratory for HIV/AIDS Clinical Research (LaPClin) is situated within the Instituto Nacional de Infectologia Evandro Chagas (INI, formerly Instituto de Pesquisa Clínica, IPEC) of the Oswaldo Cruz Foundation (FIOCRUZ) in Rio de Janeiro, Brazil. INI is a national reference center for infectious diseases and LaPClin has been a reference center for care, research, and training related to HIV/AIDS since 1986. LaPClin is one of the largest providers of primary, specialty, and tertiary care for HIV-infected individuals in the state of Rio de Janeiro.

The INI HIV Clinical Cohort is a seroprevalent cohort with follow-up since the start of the AIDS epidemic in Brazil in 1986. Since then, over 5,500 HIV infected individuals have received care at INI. An observational, longitudinal, clinical database was established in 1998 (when all patients seen from 1986 to 1998 were retrospectively included) and is updated for all patients receiving HIV and specialty care (including cardiology, endocrinology, ophthalmology, dermatology, gastroenterology, gynecology and proctology) at INI. The INI HIV Clinical Cohort is was approved by the ethics committee of the Evandro Chagas Clinical Research Institute, Oswaldo Cruz Foundation. Participants provided written informed consent.

The database is comprehensive in that it includes demographic, behavioral, clinical, laboratory, and therapeutic information (including the prescription of antiretroviral drugs)

abstracted from the medical records of patients. Patients are seen by the medical provider as recommended by the Brazilian HIV treatment guidelines. The medical record was paper-based until 2004 and is currently in an electronic format. Trained abstractors record all information onto standardized forms. Longitudinal databases include laboratory results (including hemoglobin, CD4/CD8, and HIV viral load counts, among others), types and dates of prophylaxis use, serology for hepatitis B and C among others, vaccinations, and AIDS-related and non-AIDS related diagnosis (both outpatient and inpatient). Longitudinal information regarding antiretroviral therapy prescribed includes start and stop dates for each drug of each regimen. When a new patient is included in the cohort, efforts are made to ensure the collection of information for the time prior to enrollment, including HIV-related diagnoses, laboratory results, and antiretroviral treatment.

Information regarding vital status is checked using the patients' medical charts, through active contact with individuals and family members, and by linkage with the Rio de Janeiro mortality database using a previously validated algorithm (2). Additionally, causes of death are reported according to the procedures and guidelines of the CoDe Project. The Coding Causes of Death in HIV (CoDe) Project is a standardized method for coding the underlying, immediate and contributing causes of death in HIV-infected populations (3). Taken together, these procedures allow us to confidently report on mortality and causes of death for our cohort. Descriptions of the cohort procedures and results have been published elsewhere (4-7).

Collection of INI Cohort Data for the CEPAC Model

Cohort characteristics

To estimate the characteristics of the individuals who currently seek and enroll into HIV care and initiate ART in Brazil, the study population included ART-naïve adult patients (age ≥ 18 years) who enrolled in the cohort and had a minimum follow-up of 60 days, from January 01, 2000 through December 31, 2014 (2,290 patients). Mean age at treatment initiation varied minimally by era (36.8, 38.1, 37.0, 36.0, 34.8 years in Eras 2 through 6) and was assumed constant; mean (SD) age at treatment initiation was 37 (10) years.

Similarly, gender distribution did not significantly vary by era (63%, 71%, 71%, 73%, 72% in Eras 2 through 6); overall 70% of patients were male.

CD4 counts at treatment initiation were defined within a window of 90 days of treatment start date, and if more than one measurement was available, the value closest to treatment start date was chosen. HIV viral load at treatment initiation were defined within 180 days prior to treatment start date, and if more than one measurement was available, the value closest to treatment start date was chosen. We also estimated the proportion of patients initiating ART with a history of opportunistic infections, defined as the presence of a CDC 1993 AIDS defining illness prior to or up to 30 days after treatment initiation.

A linear model was created using mean CD4 at ART initiation for patients initiating ART between 2002 and 2013 (years 2000 and 2001 were excluded from the model due to low numbers, Figure S1). Using this model, we extrapolated backwards through 1997. The average of the predicted mean CD4 counts in the years comprising each era was used as model input. Given the dramatic policy change in Era 6 (2014) specifying ART eligibility for all HIV-infected patients in Brazil, the increased mean CD4 point estimate of 518 cells/μL from the INI cohort data was used instead of an extrapolated value from the model.

Similarly, to estimate the proportion of patients initiating ART with a history of opportunistic infections, we fitted a linear model to the logistic transformation of the proportion and back transformed the predicted values to the original scale (Figure S2). Data from patients starting ART from 2002 to 2013 were used to create the model, and we extrapolated backwards to the year 1997. The average of the predicted proportion of patients initiating ART with a history of OI in the years comprising each era was used as model input. Given the dramatic policy change in Era 6 (2014) specifying ART eligibility for all HIV-infected patients in Brazil, the decreased proportion of patients initiating ART with a history of opportunistic infection of 13% from the INI cohort data was used instead

of an extrapolated value from the model. In the CEPAC model, patients with a history of opportunistic infection experience higher rates of mortality and poorer clinical outcomes.

Natural History

For the natural history parameters, the study population included adult patients (age ≥ 18 years) who enrolled in the INI cohort and had a minimum follow-up of 60 days from September 12, 1986 (the date of the first enrollee) through December 31, 2010. In order to adequately estimate parameters that could populate the CEPAC-International Model, a patient's follow-up time was stratified into the following broad categories of states: Acute Disease, Chronic HIV, and Death.

The Chronic HIV Health State consists of the time contributed by each patient stratified by CD4 count strata. The following CD4 count strata were considered: >500/µl, 301–500/µl, 201–300/µl, 101–200/µl, 51–100/µl, or <50/µl. Each patient could contribute time to either one or multiple time periods within each CD4 stratum. ~~Linear interpolation between two known CD4 measures was implemented to estimate the time points when a patient crossed the boundary between CD4 strata.~~ Time contributed to the Chronic HIV category was censored in two different situations. First, for patients who were diagnosed with a specific AIDS-defining event, follow-up was censored for both the month preceding and the two months following an opportunistic illness diagnosis. Second, for patients who died, follow-up was censored at one month prior to the date of death. Whenever possible, follow-up time was further stratified by use of antiretroviral therapy yielding periods on ART and off ART. That is, patients could contribute a certain portion of their follow-up time to an off-ART period and a certain portion of their follow-up time to an on-ART period. Also, whenever possible, separate analyses were performed for patients with and without a documented history of any AIDS-defining illness, defined by the 1993 CDC definitions (8), at cohort entry. The following AIDS-defining illnesses were specifically evaluated: Pneumocystis jirovecii pneumonia, Mycobacterium avium complex, toxoplasmic encephalitis, cytomegalovirus, tuberculosis, and other AIDS-defining events.

The Acute Disease Health State was defined by the time period from one month prior to two months after the diagnosis of a specific AIDS-defining illness. Thus, it was defined only for patients who had an AIDS-defining event(s) during the follow-up time. Time contributed to the this health state was censored before the end of the two months after illness diagnosis if the patient died, in which case the time contributed was counted for the Death Health State. The Death Health State was the period of time defined by the one month preceding the date of death.

Incidence rate of opportunistic infections

The occurrence of an opportunistic infection was defined for the first occurrence of a CDC 1993 disease. For patients who never experienced an opportunistic infection, end of follow-up was defined as the earlier of death or December 31, 2010 when administrative censoring was applied. Opportunistic infections were stratified into four groups according to their frequency: tuberculosis, toxoplasmosis, PCP+MAC+CMV, and others. To classify opportunistic infections by CD4 stratum in the INI cohort, we linearly interpolated between known CD4 measures to estimate the CD4 at which (and stratum in which) the infection occurred. The incidence rates were calculated by dividing the number of opportunistic infections by the sum of the follow-up time for all individuals in each of the Chronic HIV Health States. Additional estimates included the incidence rate of opportunistic infections stratified by the use of antiretroviral therapy when events and follow-up time were divided into periods when a specific patient had not yet started ART and after the start of ART. Incidence rates were assumed to remain constant over the time period evaluated and converted into monthly probabilities for model input (Table 1).

Mortality rates

Start of follow-up was defined as the date of cohort enrollment and end of follow-up was defined as the earlier of either death or December 31, 2010 when administrative censoring was applied. Mortality rates were then calculated by dividing the number of deaths by the sum of the follow-up time of each individual in the Chronic HIV Health State. Additional estimates included the mortality rate stratified by the use of antiretroviral therapy and by

the history of prior opportunistic infection, defined as an opportunistic infection occurring prior to cohort enrollment. Mortality rates were assumed constant for the time period evaluated and converted into monthly probabilities for model input.

Loss to Follow-up (LTFU) and Return to Care (RTC)

LTFU rates were ascertained from the HIV-Brazil Cohort Study, which has followed a cohort of over 5,000 patients across 26 public health care facilities since 2002. In this study, it was possible to track transfers between public health facilities such that patients who were transferred to other facilities were not considered lost to follow-up. The reported average rate of loss to follow-up was 10.1/1000PY (9). Patients with adherence values greater than 95% were subject to a rate of loss to follow-up of 4.0/1000PY, and those with adherence values less than 50% experienced a rate of loss to follow-up of 27.6/1000PY, linearly interpolating for adherence values between 50-95%, to arrive at the average value of 10.1/1000PY.

For the subset of patients who were lost to follow-up, a rate of RTC was calculated using INI data as the number of patients who returned to care divided by the total person-years lost to follow-up. This rate of 818/1000PY was assumed for all patients lost to follow up, regardless of adherence level.

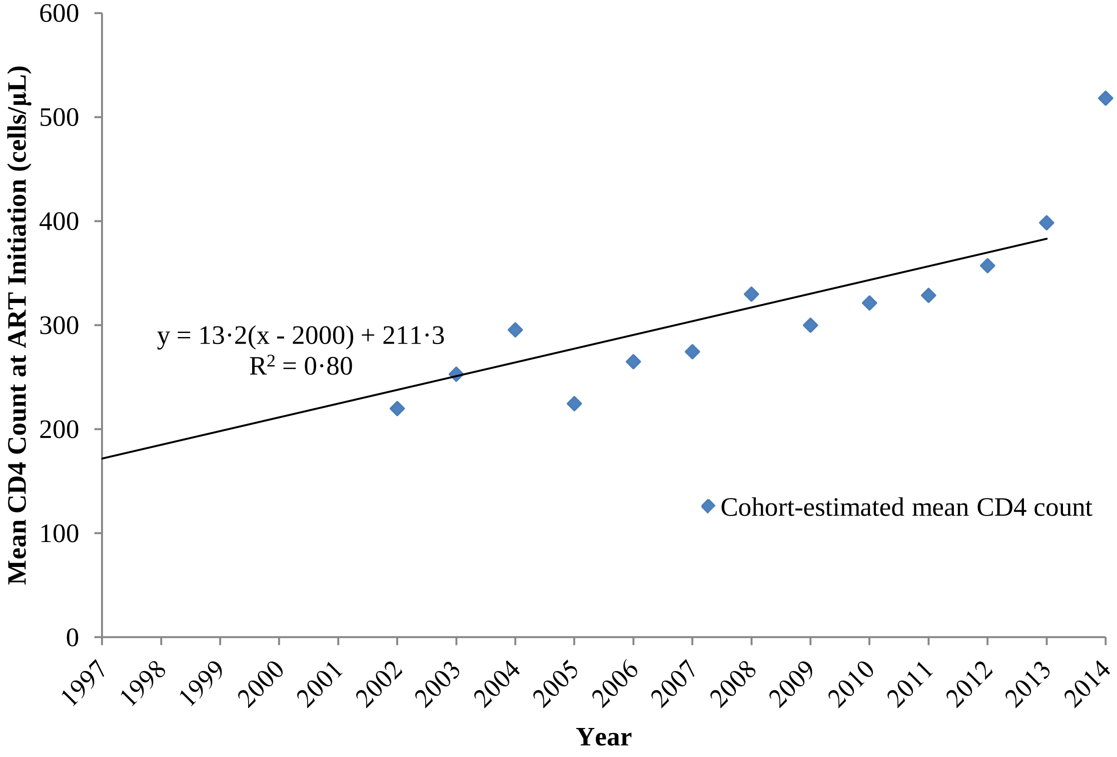

Figure S1. Mean CD4 count at ART initiation in INI cohort, 2002-2014, and fitted linear model (excluding 2014)

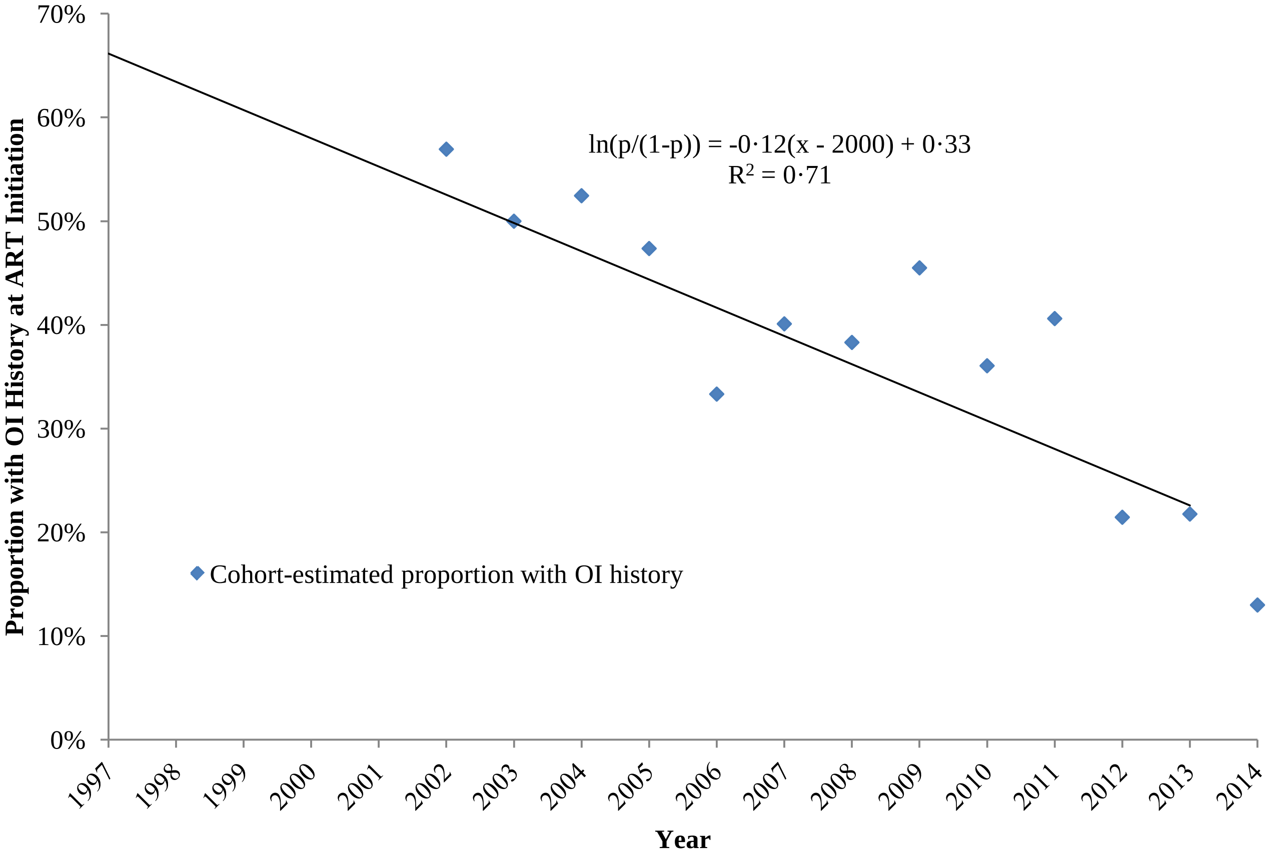

Figure S2. Percent of patients with OI History at ART initiation in INI cohort, 2002-2014, and fitted logistic model (excluding 2014)

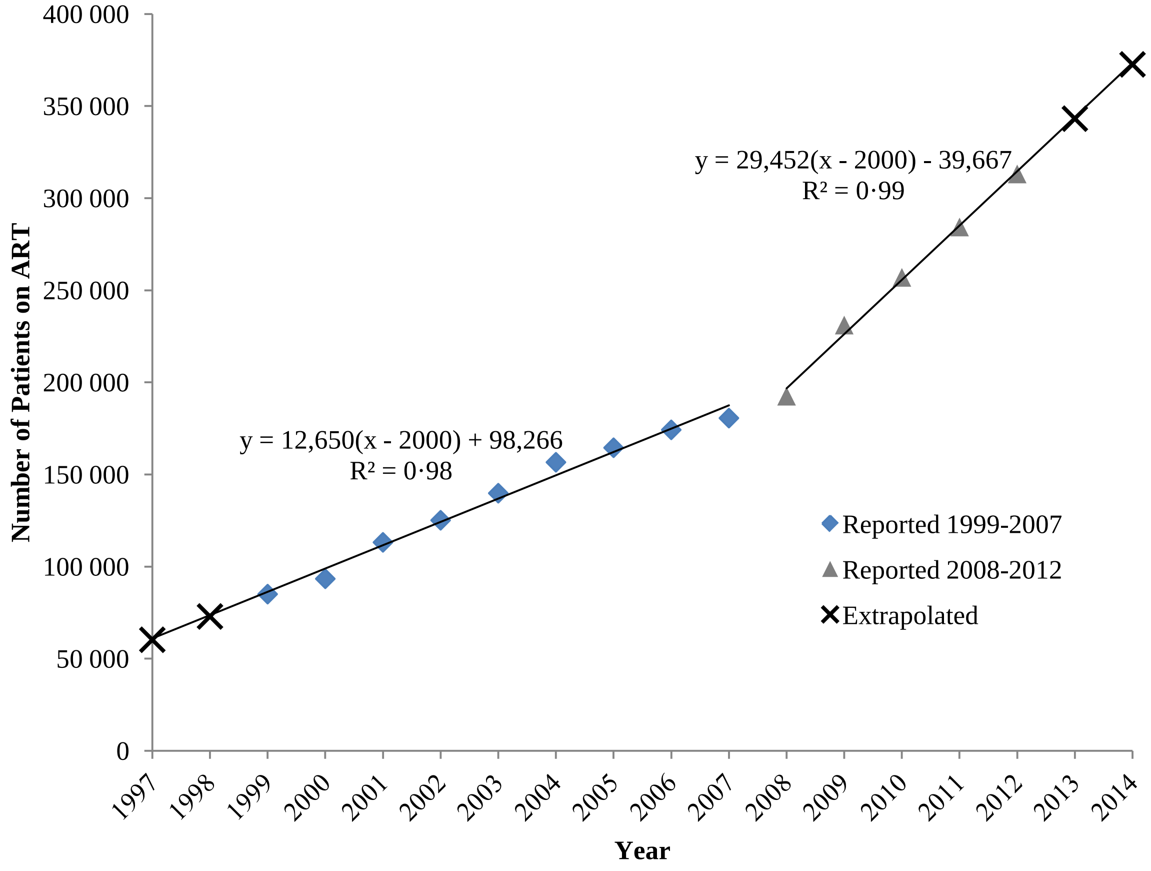

Figure S3. Patients Receiving Antiretroviral Therapy in Brazil, Brazil Ministry of Health Boletim Epidemiologico HIV/AIDS 2011

Table S1. Selected Latin American countries and respective demographic, economic, and HIV statistics

**Country**	**Population (millions)**	**HIV Prevalence (%)(8, 10)**	**Estimated new HIV infections in 2013 (age 15+)(8)**	**2013 Per Capita GDP (USD)(11)**	**Type of Epidemic**	**ART Program**	**Proportion of eligible HIV-infected individuals on ART (%)(8)**	**Number of patients on ART, 2012(8)**	**Domestic HIV spending from domestic public sources, 2010 (millions USD)(8)**
**Brazil**	203.6	0.6	44,000	11,200	Concentrated	National, free of charge (1996)	87	307,025	745.8
**Argentina**	41.4	0.4	5,100	14,700	Concentrated	National, free of charge (1997) (12)	81	47,725	Not available
**Chile**	16.8	0.3	2,100	15,700	Concentrated	National, free of charge (2005) (13)	86	18,898	119.2
**Colombia**	47.9	0.5	8,400	7,800	Concentrated	National, subsidized (2004) (14)	51	33,148	87.0
**Ecuador**	15.9	0.4	2,500	6,000	Concentrated	National, subsidized (2002) (15)	42	9,080	24.3
**Mexico**	119.7	0.2	9,200	10,300	Concentrated	National, free of charge (2001) (12)	82	82,000	Not available
**Peru**	30.5	0.4	3,400	6,700	Concentrated	National, free of charge (2004) (16)	60	27,007	15.4

**Venezuela**	30.2	0.6	6,000	14,400	Concentrated	National, free of charge (2000) (17)	72	42,060	109.0
**Latin America**	588.0	0.4	92,000	-	Concentrated	-	75	619,104	-

Note: 'National' is used to denote payment by a federal body

REFERENCES

1. Mellors JW, Munoz A, Giorgi JV, Margolick JB, Tassoni CJ, Gupta P, et al. Plasma viral load and CD4+ lymphocytes as prognostic markers of HIV-1 infection. Annals of internal medicine. 1997 Jun 15;126(12):946-54. PubMed PMID: 9182471.

2. Pacheco AG, Saraceni V, Tuboi SH, Moulton LH, Chaisson RE, Cavalcante SC, et al. Validation of a hierarchical deterministic record-linkage algorithm using data from 2 different cohorts of human immunodeficiency virus-infected persons and mortality databases in Brazil. American journal of epidemiology. 2008 Dec 1;168(11):1326-32. PubMed PMID: 18849301. Pubmed Central PMCID: 2638543.

3. Kowalska JD, Friis-Moller N, Kirk O, Bannister W, Mocroft A, Sabin C, et al. The Coding Causes of Death in HIV (CoDe) Project: initial results and evaluation of methodology. Epidemiology. 2011 Jul;22(4):516-23. PubMed PMID: 21522013.

4. Campos DP, Ribeiro SR, Grinsztejn B, Veloso VG, Valente JG, Bastos FI, et al. Survival of AIDS patients using two case definitions, Rio de Janeiro, Brazil, 1986-2003. Aids. 2005 Oct;19 Suppl 4:S22-6. PubMed PMID: 16249649.

5. Grinsztejn B, Luz PM, Pacheco AG, Santos DV, Velasque L, Moreira RI, et al. Changing mortality profile among HIV-infected patients in Rio de Janeiro, Brazil: shifting from AIDS to non-AIDS related conditions in the HAART era. PloS one. 2013;8(4):e59768. PubMed PMID: 23577074. Pubmed Central PMCID: 3618173.

6. Grinsztejn B, Veloso VG, Friedman RK, Moreira RI, Luz PM, Campos DP, et al. Early mortality and cause of deaths in patients using HAART in Brazil and the United States. Aids. 2009 Oct 23;23(16):2107-14. PubMed PMID: 19770698. Pubmed Central PMCID: 3790467.

7. Moreira RI, Luz PM, Struchiner CJ, Morgado M, Veloso VG, Keruly JC, et al. Immune status at presentation for HIV clinical care in Rio de Janeiro and Baltimore. Journal of acquired immune deficiency syndromes. 2011 Aug;57 Suppl 3:S171-8. PubMed PMID: 21857314. Pubmed Central PMCID: 3170137.

8. UNAIDS. The Gap Report 2014 [cited 2015 15 April]. Available from: http://www.unaids.org/sites/default/files/media_asset/UNAIDS_Gap_report_en.pdf.

9. Grangeiro A, Escuder MM, Cassanote AJ, Souza RA, Kalichman AO, Veloso V, et al. The HIV-Brazil cohort study: design, methods and participant characteristics. PloS one. 2014;9(5):e95673. PubMed PMID: 24789106. Pubmed Central PMCID: 4006775.

10. UNAIDS. The Global Report 2013 [cited 2015 15 April]. Available from: http://www.unaids.org/sites/default/files/media_asset/UNAIDS_Global_Report_2013_en_1.pdf.

11. The World Bank. GDP per capita (current US$) 2013 [cited 2015 15 April]. Available from: http://data.worldbank.org/indicator/NY.GDP.PCAP.CD/countries/1W?display=default.

12. Bertozzi S, Padian NS, Wegbreit J, DeMaria LM, Feldman B, Gayle H, et al. HIV/AIDS Prevention and Treatment. In: Jamison DT, Breman JG, Measham AR, Alleyne G, Claeson M, Evans DB, et al., editors. Disease Control Priorities in Developing Countries. 2nd ed. Washington (DC)2006.

13. Comision Nacional del SIDA. Estrategia de atencion integral a personas que viven con VIH/SIDA programa nacional de prevencion y control del VIH/SIDA 2007 [cited 2015 15 April]. Available from: http://www.aidstar-one.com/sites/default/files/prevention/resources/national_strategic_plans/Chile_2007.pdf.

14. Ministerio de la Protección Social Dirección General de Salud Pública. Plan Nacional de Respuesta Ante el VIH y el SIDA Colombia 2008-2011 2008 [cited 2015 15 April]. Available from:

http://www.aidstar-one.com/sites/default/files/prevention/resources/national_strategic_plans/Colombia_2008-2011.pdf.

15. Ministerio de Salud Pública del Ecuador. Plan Estratégico Multisectorial de la Respuesta Nacional al VIH/SIDA 2007-2015 2007 [cited 2015 15 April]. Available from: http://www.aidstar-one.com/sites/default/files/prevention/resources/national_strategic_plans/Ecuador_2007-2015.pdf.

16. Caceres CF, Mendoza W. The national response to the HIV/AIDS epidemic in Peru: accomplishments and gaps--a review. Journal of acquired immune deficiency syndromes. 2009 May 1;51 Suppl 1:S60-6. PubMed PMID: 19384104.

17. Pearson T. Venezuelan Government Continues to Provide Free HIV and AIDS Treatment 2014 [cited 2015 15 April]. Available from: http://venezuelanalysis.com/news/10312.
